# Supraspinatus extraction from MRI based on attention-dense spatial pyramid UNet network

**DOI:** 10.1186/s13018-023-04509-7

**Published:** 2024-01-13

**Authors:** Peng Wang, Yang Liu, Zhong Zhou

**Affiliations:** 1 Third Clinical Medical School, Nanjing University of Chinese Medicine, Nanjing, 210023 People’s Republic of China; 2 Affiliated Hospital of Integrated Traditional Chinese and Western Medicine, Nanjing University of Chinese Medicine, No. 100 Maigaoqiao Cross Street, Qixia District, Nanjing City, 210028 Jiangsu Province People’s Republic of China; 3 School of Remote Sensing and Geomatics Engineering, Nanjing University of Information Science & Technology, Nanjing, 210044 People’s Republic of China

**Keywords:** Deep learning, Medical imaging, MRI, Rotator cuff, Supraspinatus extraction

## Abstract

**Background:**

With potential of deep learning in musculoskeletal image interpretation being explored, this paper focuses on the common site of rotator cuff tears, the supraspinatus. It aims to propose and validate a deep learning model to automatically extract the supraspinatus, verifying its superiority through comparison with several classical image segmentation models.

**Method:**

Imaging data were retrospectively collected from 60 patients who underwent inpatient treatment for rotator cuff tears at a hospital between March 2021 and May 2023. A dataset of the supraspinatus from MRI was constructed after collecting, filtering, and manually annotating at the pixel level. This paper proposes a novel A-DAsppUnet network that can automatically extract the supraspinatus after training and optimization. The analysis of model performance is based on three evaluation metrics: precision, intersection over union, and Dice coefficient.

**Results:**

The experimental results demonstrate that the precision, intersection over union, and Dice coefficients of the proposed model are 99.20%, 83.38%, and 90.94%, respectively. Furthermore, the proposed model exhibited significant advantages over the compared models.

**Conclusion:**

The designed model in this paper accurately extracts the supraspinatus from MRI, and the extraction results are complete and continuous with clear boundaries. The feasibility of using deep learning methods for musculoskeletal extraction and assisting in clinical decision-making was verified. This research holds practical significance and application value in the field of utilizing artificial intelligence for assisting medical decision-making.

## Background

The rotator cuff is composed of the subscapularis, supraspinatus, infraspinatus, and teres minor tendons. This structure connects the scapula to the humeral head and maintains dynamic stability of the glenohumeral joint through a concave compression mechanism. It is also an essential component in maintaining the equilibrium of couples in the shoulder joint [1]. Rotator cuff tears (RCTs) can cause pain and limitation of shoulder motion, with the supraspinatus tendon being the most commonly affected site. A recent study found that RCTs account for approximately 50%–85% of shoulder disorders treated by clinicians, and the morbidity increases with age [2].

The rotator cuff tendinopathy has been classically summarized as extrinsic, intrinsic, or a combination of both. Intrinsic mechanisms, such as the mechanical properties, age-related degeneration, and vascularity of the rotator cuff, along with extrinsic mechanisms such as internal and external impingement caused by alterations in scapular and glenohumeral kinematics, appear to be significant contributors to RCTs [3]. Tears most commonly occur in and around the critical zone of the supraspinatus tendon, which lies in the region between the bony insertion of the tendon and the nearest musculotendinous junction [4]. This anatomic factor combined with multiple internal and external mechanisms contributes to this result, such as the morphology of supraspinatus [5], subacromial impingement [6], and the presence of “critical zone” [7, 8]. The high incidence of supraspinatus tear gives its segmentation a higher priority and considerable clinical significance for the diagnosis of RCTs.

In clinical practice, shoulder magnetic resonance imaging (MRI) plays a crucial role in diagnosing RCTs, assessing the extent of tears, and formulating surgical plans. MRI offers advantages such as non-invasiveness, non-ionizing, anatomical reproducibility, and excellent tissue contrast, making it a common modality for the clinical diagnosis of RCTs and preoperative preparation [9].

Currently, computer-aided diagnosis (CAD) techniques have been widely applied in medical image analysis, significantly enhancing diagnostic accuracy and efficiency. With the advent of the artificial intelligence (AI) revolution, AI-enabled health care has become a hot research field. Targeting the issues of low efficiency in the interpretation of massive MRI images and subjective differences in human interpretation, this paper proposes deep learning (DL) methods and builds an innovative DL model based on existing research findings. It was developed to automatically segment and extract regions of interest, aiming to alleviate the workload of clinical doctors.

The supraspinatus is the most common site of RCTs, and its tears along with atrophy can reflect the severity of damage. Therefore, the supraspinatus is chosen as the segmentation target, and an improved DL model that can accurately extract the supraspinatus in the coronal plane was proposed in this paper. Compared to the more extensively studied sagittal plane, the coronal plane is a vertical plane perpendicular to the body. It is commonly used to observe the anterior–posterior thickness and morphology of the supraspinatus muscle in the shoulder. The coronal plane is particularly useful in assessing tears or changes in the anterior–posterior thickness of the supraspinatus muscle. Therefore, compared to segmentation based on the sagittal plane, extracting the supraspinatus muscle based on the coronal plane can improve the efficiency of diagnosing and treating RCTs. It has a significant impact on clinical decision-making and the formulation of surgical plans. This innovation holds certain practical significance in the field of intelligent recognition and interpretation of medical images.

## Methods

**Fig. 1:**
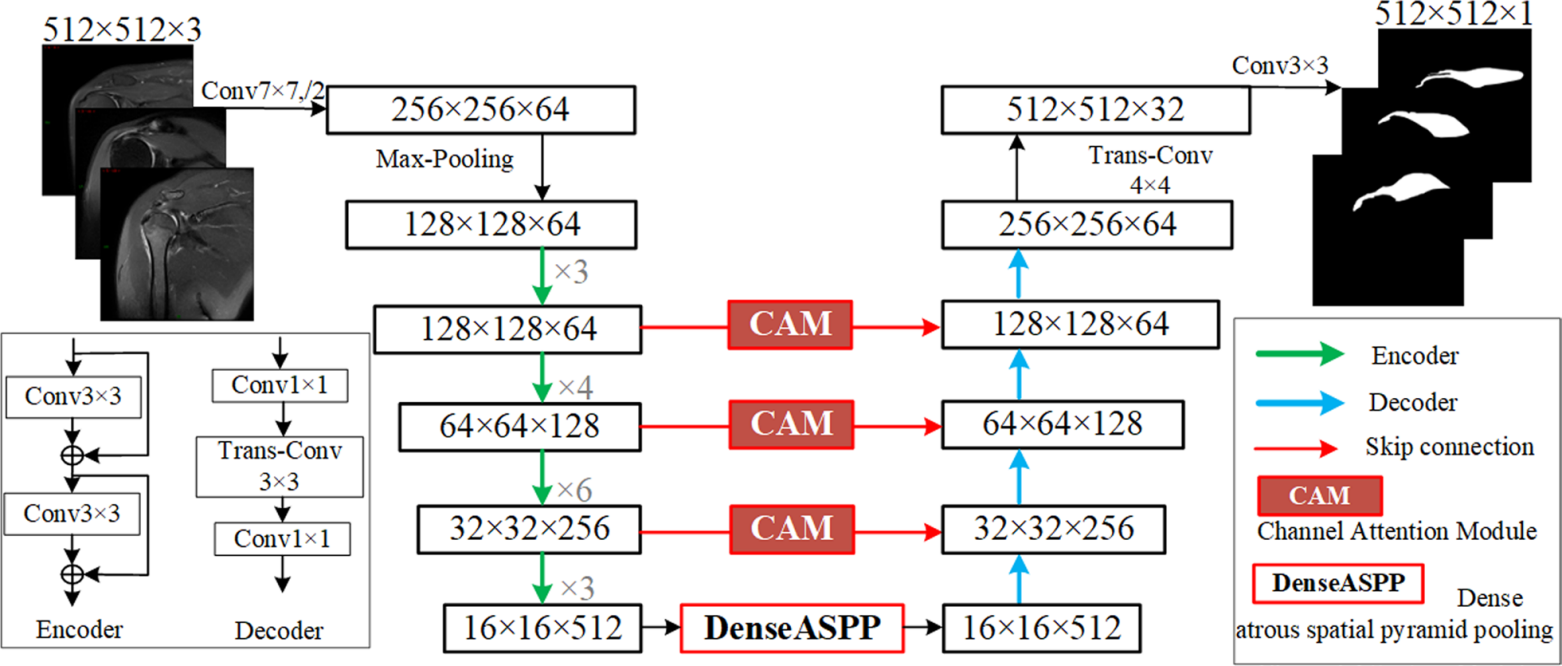
Improved model architecture diagram based on LinkNet

Shoulder MRI has relatively simple semantics and fixed structures. Both high-level and low-level semantic information are equally important, and there is a high demand for timeliness in medical diagnosis. Therefore, image segmentation algorithms are commonly used in research to improve segmentation results and accuracy while reducing manual intervention and segmentation time. Considering these characteristics, this paper chooses the LinkNet [10] model as the base framework. LinkNet has demonstrated good performance in achieving accurate segmentation results. It utilizes a combination of encoder and decoder structure to capture local and global context information, which is crucial for accurately segmenting the supraspinatus muscle and distinguishing it from surrounding tissues. LinkNet strikes a balance between accuracy and efficiency based on a lightweight network structure. It computes efficiently while still maintaining competitive segmentation performance. This is especially valuable in clinical settings for real-time or near real-time MRI image analysis. The LinkNet shows robustness in handling image quality and noise variations. The model’s structure helps mitigate the impact of noise and artifacts, resulting in more reliable segmentation results, showing. This robustness is essential for accurate segmentation of the supraspinatus muscle. And LinkNet can be pre-trained on large datasets, such as medical image repositories, to enhance model generalization and improve segmentation performance. Considering these factors, the LinkNet model is a suitable choice for accurate and efficient supraspinatus muscle segmentation in MRI images. However, the MRI images of the supraspinatus encompass intricate details and local features, accurate segmentation is challenging, and the selection and improvement of any segmentation model depends on the specific requirements of the task. This paper constructs an attention-dense atrous spatial pyramid pooling UNet (A-DAsppUnet) network for the segmentation of the supraspinatus in shoulder MRI. As shown in Fig. 1, the proposed model involves an encoder ResNet34 [11], a channel attention module, and dense atrous spatial pyramid pooling (DenseASPP), which connects the encoder and decoder network. The encoder can extract deep semantic feature information, while the channel attention incorporates skip connections to enhance feature representation during encoding and decoding. Drawing from the structural experience of the D-LinkNet model [12], DenseASPP is beneficial to capture multi-context information, intensive feature extraction, and parameter sharing and improves the accuracy of semantic segmentation. It is widely used in object segmentation [13] and scene semantic recognition [14, 15]. The aforementioned structures were integrated into the model and had been innovatively applied to muscle tissue segmentation in medical MRI images. The proposed model demonstrates the ability to resist noise and image quality interference, enabling efficient and accurate segmentation of the supraspinatus and surrounding tissues.

As shown in the diagram, the selected sequences were downloaded and exported as TIFF files and saved across three RGB image channels, which were adjusted to 8-bit 512 × 512 × 3-pixel portable network graphics (PNG) files using Photoshop to match the standardized network input. Compared with the original grayscale image, which only contains brightness information, RGB image helps to separate the supraspinatus muscle from the surrounding tissue. Due to the inclusion of three information channels, RGB images facilitate the differentiation of certain muscle diseases or conditions that may result in color variations in muscle tissue. Moreover, the richness of contextual information in RGB images enhances their visualization effect, making them more intuitive and suitable for specific algorithms. The ResNet34 model utilizes multiple down-sampling steps to extract the desired target features. At the end of the encoding process, the image dimensions are reduced to a size of 16 × 16 with 512 channels. Subsequently, the feature map is passed into the DenseASPP module. This step is beneficial for expanding the receptive field without compromising the resolution of the feature map. Additionally, it ensures the preservation of abundant spatial information. In the decoding stage, the feature map size is restored through transposed convolution for up-sampling. The model utilizes skip connections and channel attention modules to fuse and complement the feature information, enhancing both the integrity of the feature information and the exchange of channel features. This approach significantly improves the network's capability to extract target regions in complex MRI images, ensuring high accuracy and robustness in feature extraction.

### Channel attention module

**Fig. 2:**
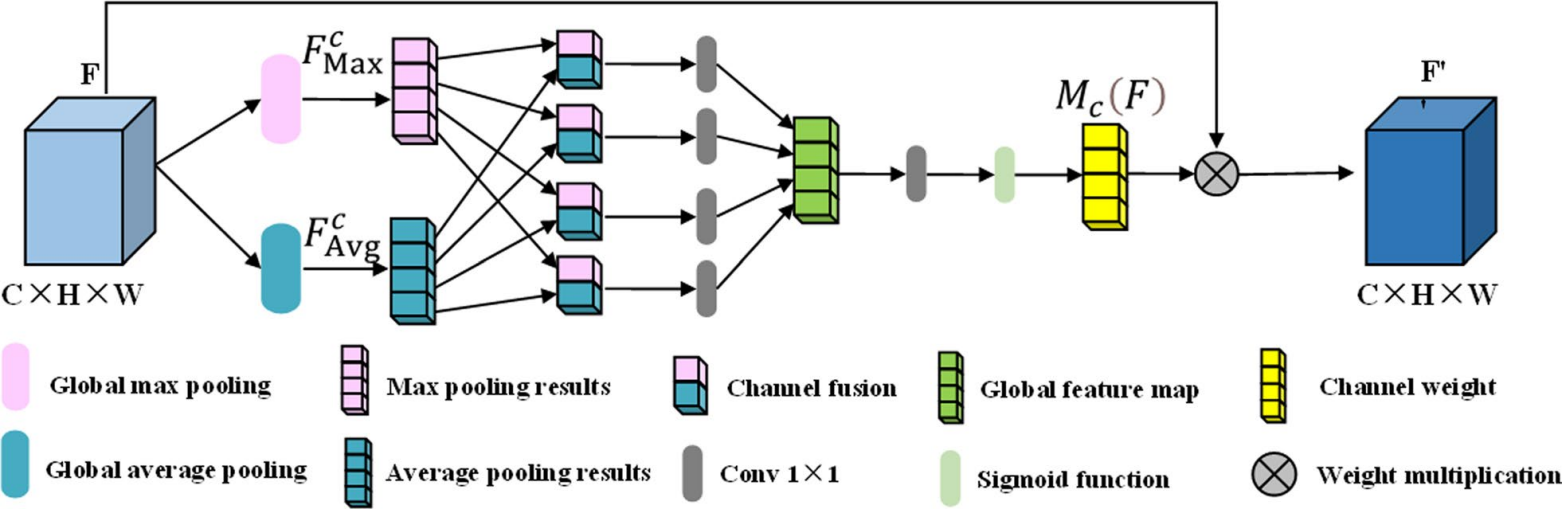
Channel attention diagram [18]

In medical image segmentation, extracting structural features of target regions is often challenging. Additionally, the performance and stability of medical image segmentation models are often compromised due to the lack of high-quality manually labeled datasets and class imbalance among the samples. Attention mechanisms have demonstrated their effectiveness in enhancing a model's ability to focus on important features [16]. In this paper, a channel attention mechanism is introduced to adjust the importance of each channel in the feature maps after encoding and down-sampling. This mechanism dynamically adjusts the network's attention to different features, thereby effectively enhancing feature extraction and utilization. It helps alleviate class imbalance issues, reduce noise, and eliminate redundant information. As a result, it improves the robustness of the model and enhances segmentation accuracy [17]. Figure 2 illustrates the structure of the channel attention module.

### Dense atrous spatial pyramid pooling

**Fig. 3:**
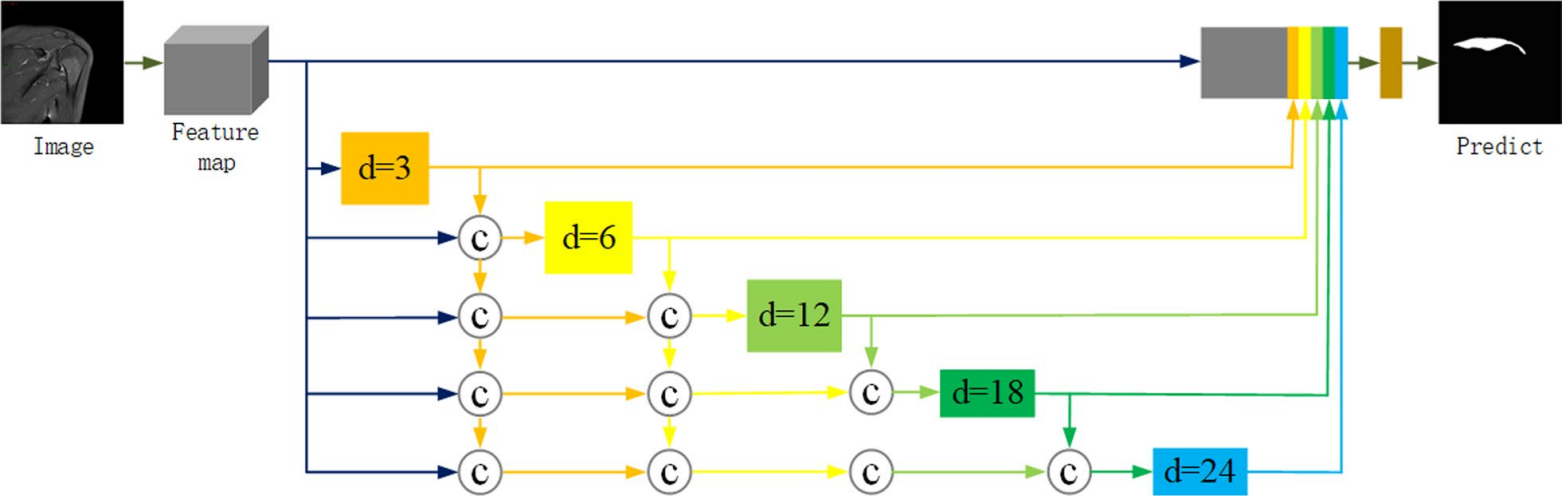
DenseASPP structure diagram [15]

In the middle part of the model, the DenseASPP structure based on the dense convolutional network (DenseNet) [19] model is used to connect the encoding and decoding networks. Figure 3 shows the structure of DenseASPP, it utilizes multiple branches of different void convolution kernels to extract multi-scale features from the input data. In medical image segmentation research, accurate segmentation of the target region is a crucial performance metric. The DenseASPP module expands the receptive field and adapts to multi-scale input images by utilizing dilated convolutions and pyramid pooling within a dense block structure. In Fig. 3, d represents the dilation rate of dilated convolutions. This module enhances the semantic expressive power of features and exhibits outstanding performance in image segmentation tasks.

## Experiment and analysis

### Experimental data and comparative models

#### Experimental data

This paper was approved by the Ethics Committee of Jiangsu Province Hospital with Integration of Chinese and Western Medicine, and the approval number is 2023-LWKYZ-033. Personal informed consent was waived for this retrospective study. It retrospectively collected data from 60 patients who underwent inpatient treatment for RCTs at the hospital between March 2021 and May 2023. Patients with other shoulder conditions, such as fractures, dislocations, and calcific tendinitis, were excluded from the study.

Examinations were acquired with a 1.5 T MR scanner (General Electric, SIGNA CREATOR). Conventional two-dimensional MR images were obtained from the proton density (PD) fat-suppressed sequence in the oblique coronal plane. The acquisition parameters are as follows: TR = 2278 + ms, TE = 12.6–84.2 ms, FOV = 20 cm, NEX = 2, bandwidth = 31.25 Hz/pixel, slice thickness = 4 mm, and spacing: 0.5 mm.

Due to the physiological differences between individuals and the diverse location of tears, the author selected images capable of displaying the supraspinatus clearly from acquired images, about 3–5 images per sequence. After the selection process, a total of 200 MRI images were chosen for further analysis. The 60 subjects were randomly divided into a training set, a validation set, and a test set, ensuring that images from the same subject in the training dataset were not used in the validation or evaluation processes in this paper. To fully train the model in this paper, the experimental data were expanded threefold using data augmentation techniques, including image rotation, horizontal flipping, and vertical flipping. These techniques aim to enhance the robustness and generalization capability of the model, thereby improving the accuracy of target extraction during model training.

**Fig. 4:**
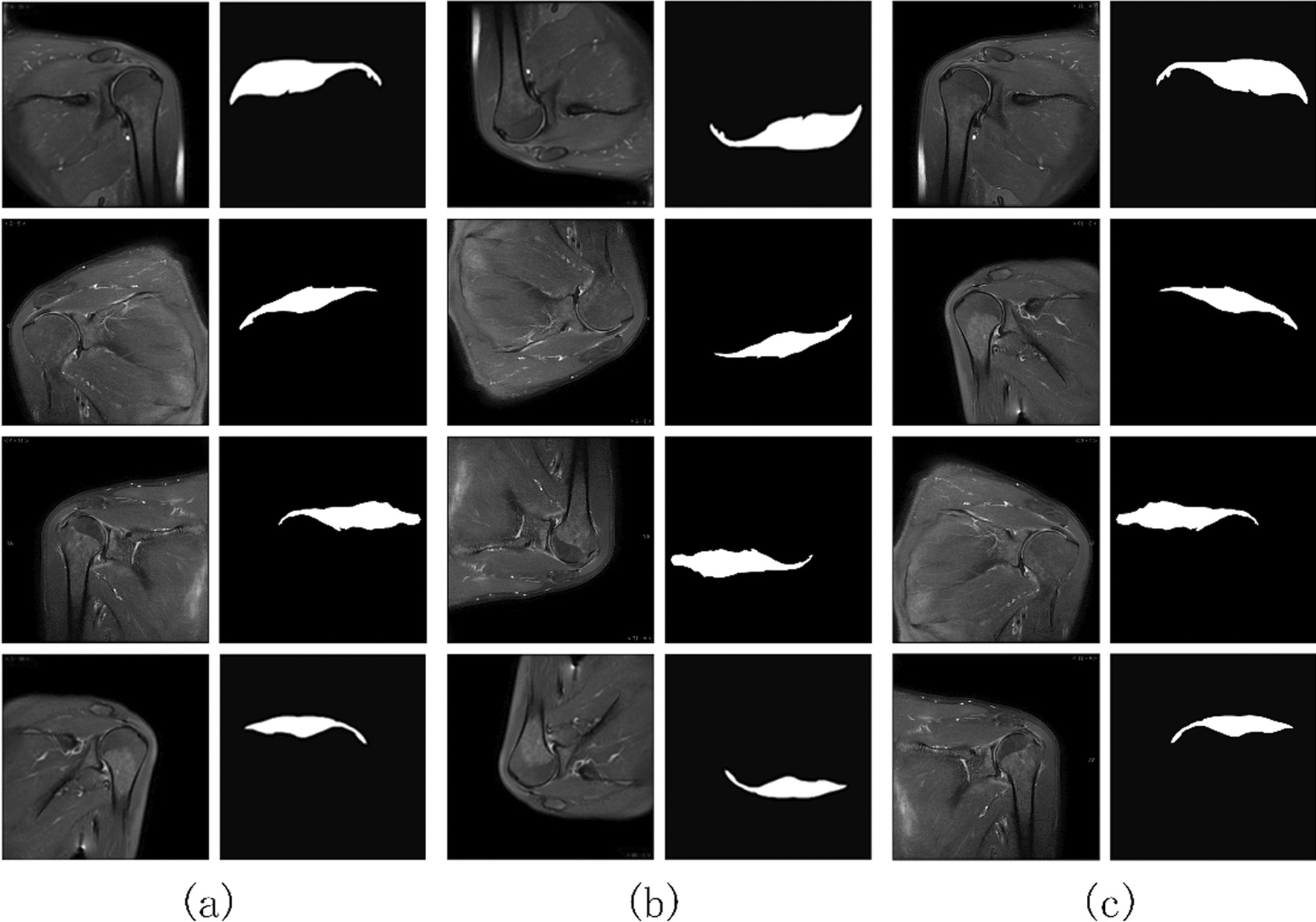
The original data and data expansion. **a** Original images and labels. **b** Rotate the image and label. **c** Flip horizontal images and labels

The images were in RGB format with a size of 512 × 512 pixels. The supraspinatus was manually annotated by tracing its contour on the images. The proximal end of the annotation started at the scapular spine, while the distal end ended at the greater tuberosity of the humerus. The superior boundary was defined by the acromion, shoulder joint capsule, and trapezius, while the inferior boundary was determined by the scapular spine, the upper aspect of the humeral head, and the supraglenoid tubercle. These annotations output corresponding labels for the supraspinatus tendon. The data were annotated by three graduate students and physicians specialized in musculoskeletal imaging. The annotations underwent verification by experienced physicians to ensure accuracy and reliability. The original images and extended data are shown in Fig. 4.

#### Comparative models

To assess the feasibility and high accuracy of the proposed model for segmenting the supraspinatus tendon in MRI images, several classic image segmentation algorithms, including fully convolution network (FCN), UNet, semantic segmentation network (SegNet), and DenseNet, were selected for comparative experiments on the dataset employed in this paper. These models were chosen to evaluate the accuracy and performance of the proposed method against established approaches.

### Experimental environment and evaluation metrics

#### Experimental environment

The experiments were conducted using the Python 3.7 programming language and the PyTorch 1.8.1 deep learning framework. All experiments were performed on a computer equipped with an AMD Ryzen 7 3700X CPU and an NVIDIA GeForce RTX 2700 graphics card with 8 GB of VRAM. For model training, the binary cross-entropy loss function (BCELoss) was used, along with the Adam optimizer to update the network parameters. The batch size was set to 2, and the learning rate was set to 0.0001. The models were trained for 30 epochs.

#### Evaluation metrics

Image segmentation is evaluated using metrics such as precision (Pre), *F*1 score, and intersection over union (IoU). However, in medical image segmentation, the Dice coefficient is often used to assess model performance. Therefore, this paper uses Pre, the Dice coefficient, and IoU as the evaluation metrics to measure model performance and the accuracy of supraspinatus tendon segmentation. The formulas for these evaluation metrics are as follows:

1 $${\text{Precision}} = \frac{{{\text{TP}}}}{{{\text{TP}} + {\text{FP}}}} \times 100\%$$

2 $${\text{IoU}} = \frac{{{\text{TP}}}}{{{\text{TP}} + {\text{FP}} + {\text{FN}}}} \times 100\%$$

3 $${\text{Dice}} = \frac{{2{\text{TP}}}}{{\left( {{\text{TP}} + {\text{FP}}} \right) + \left( {{\text{TP}} + {\text{FN}}} \right)}} \times 100\%$$

In the equations, TP represents the number of pixels correctly predicted as supraspinatus tendon, FP represents the number of pixels incorrectly predicted as supraspinatus tendon, TN represents the number of pixels correctly predicted as background, and FN represents the number of pixels incorrectly predicted as background.

### Experimental results and analysis

To ensure fair and objective analysis of the experimental results, all experiments in this paper were conducted using the same dataset and experimental environment. Table 1 displays the quantitative statistical results of the five models on the experiment's test set for supraspinatus tendon extraction. The evaluation of model performance is conducted using three assessment metrics: Pre, IoU, and Dice coefficient.

**Table 1:** Extraction result statistics

Model	Pre (%)	IoU (%)	Dice (%)
FCN	98.28	65.07	78.84
SegNet	98.53	70.59	82.76
UNet	98.74	73.61	84.80
DenseNet	98.79	75.86	86.27
A-DAsppUnet	**99.20**	**83.38**	**90.94**

Bold values indicate the performance of the proposed method over the comparison method

According to the table, the mentioned models are capable of extracting the supraspinatus tendon to some extent, with differences in terms of completeness, continuity, and accuracy of the extraction. The proposed method in this paper achieved Pre, IoU, and Dice coefficients of 99.20%, 83.38%, and 90.94%, respectively. The comparison clearly indicates that this method has a significant advantage and performs in terms of extracting the supraspinatus tendon. Compared to the four comparative models, the proposed method exhibited improvements in the evaluation metrics. The “Pre” metric showed an enhancement of approximately 0.4%–1%, the “IoU” metric witnessed an improvement of 7.5%–18.3%, and the "Dice coefficient" experienced an increase of approximately 4.7%–12.1%. These improvements were significant across all indicators. Among the comparative models, DenseNet performed the best, followed by UNet and SegNet, while FCN had the worst effect and had a large gap with the proposed algorithm in IoU and Dice indicators in this paper.

Based on the data in the table and the equation above, it can be observed that the improvement in the Pre metric is not significant. The reason is that the target pixel constitutes a relatively small proportion of the total number of pixels, and reducing false-positive pixels does not lead to a significant increase in accuracy. According to the IoU indicator and its calculation formula, a higher IoU value signifies a larger proportion of accurately classified supraspinatus pixels relative to the total number of correctly classified pixels, with fewer incorrectly predicted pixels. In the supraspinatus segmentation task, the significant improvement in the IoU measure indicates that the proposed model achieves the highest accuracy in supraspinatus segmentation. In addition, with a Dice coefficient of 90.94%, it can be inferred that the performance of the model proposed in this paper is superior.

**Fig. 5:**
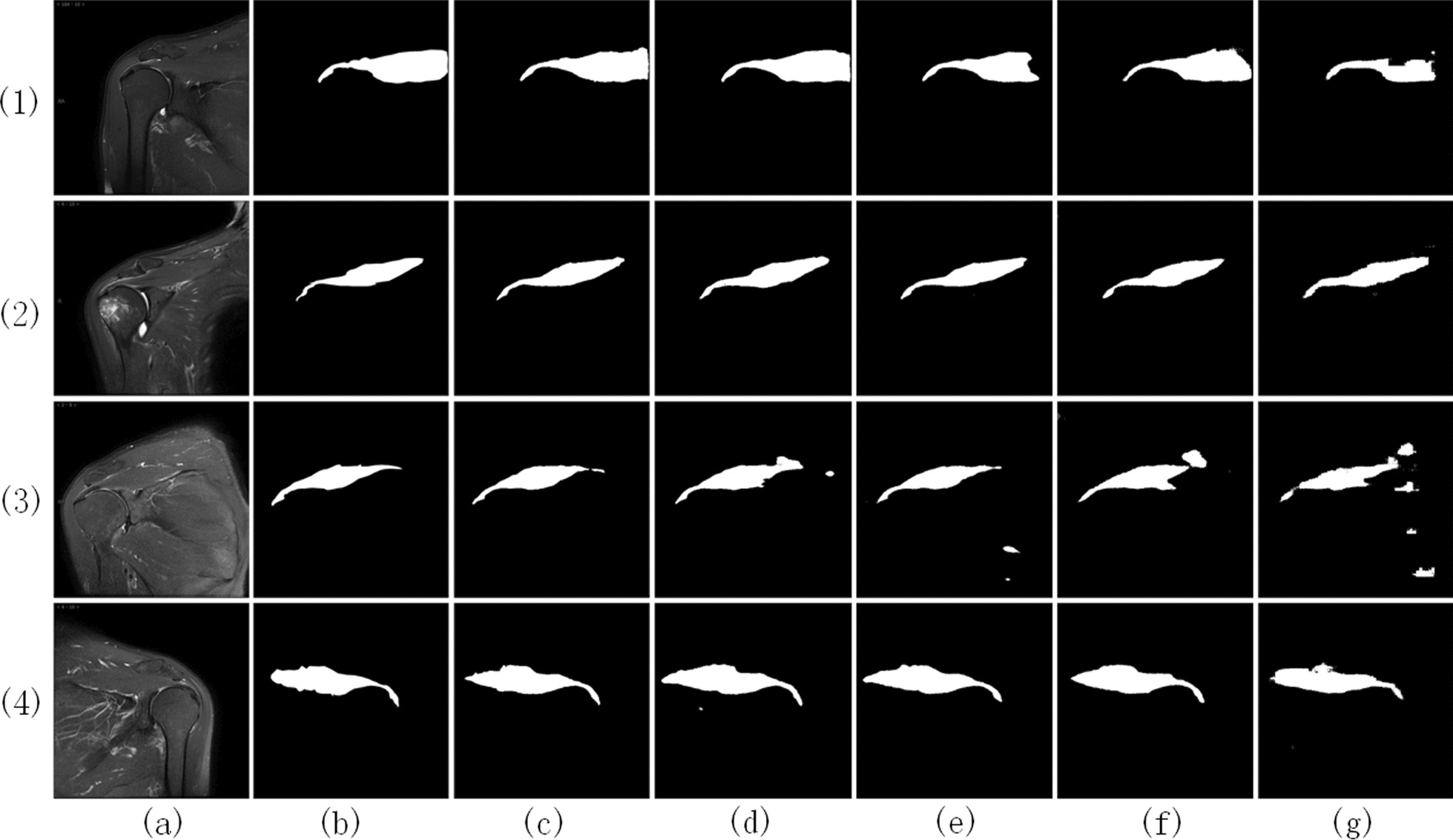
Visualization of extraction results. **a** Images. **b** Labels. **c** A-DAsppUnet. **d** DenseNet. **e** UNet. **f** SegNet. **g** FCN

To fully validate the above conclusions, this paper conducted a visual analysis of the supraspinatus segmentation results. Figure 5 illustrates a visual comparison between the results obtained using the proposed method and those obtained using the comparative model on the test set images. Four representative images are provided in the figure for comparison. As shown in the figure, the segmentation results obtained using the proposed method exhibit the best performance in terms of completeness and capturing fine details. Specifically, the extracted supraspinatus is delineated clearly from structures such as the humeral tuberosity, scapular spine, and inferior glenohumeral capsule. The segmentation results exhibit well-preserved details, and there is a high level of accuracy in aligning the upper and lower boundaries of the segmented region with the ground truth labels. In contrast, in the results obtained using the comparative model, the boundaries of the supraspinatus extraction are blurred near the side of the scapular spine in images (1) and (3). Image (1) shows poor overall segmentation performance, with a significant portion of supraspinatus pixels left unsegmented. Image (3) exhibits numerous erroneous segmentations. On the other hand, in images (2) and (4), the proposed method accurately delineated the edges of the supraspinatus, particularly at the tendon junction with the humeral head and the superior border of the deltoid muscle.

In the comparison model, DenseNet performs well in extracting the target and achieves reasonably accurate segmentation. However, it fails to capture fine details, especially in capturing the blurry boundaries with the deltoid muscle, resulting in insufficient accuracy. The UNet model lacks completeness in target extraction. For example, in image (1), there is information missing in the proximal part of the supraspinatus, and the extracted region is smaller than the actual boundaries defined by the labels. For the SegNet and FCN models, their segmentation results exhibit more false positives and false negatives, as shown in images (1) and (3).

### Robustness analysis through ablation experiments

To thoroughly validate the effectiveness of the proposed innovative model, it conducted ablation experiments to investigate the impact of the deep encoding network, channel attention, and dense spatial pyramid pooling modules on the performance of the proposed model. The experimental setup is described as follows:

LinkNet18 was selected as the baseline model in this paper. Scheme 1: ResNet34 is selected as the model encoder network. Scheme 2: Add the channel attention mechanism at the jump junction of the Scheme 1 model. Scheme 3: DenseASPP in the middle of the Scheme 1 model to connect the encoder and decoder networks. The models of the ablation experiment are shown in Table 2.

**Table 2:** Ablation experimental model

Model	ResNet34	Channel attention	DenseASPP
Baseline			
Scheme 1	√		
Scheme 2	√	√	
Scheme 3	√		√
A-DAsppUnet	√	√	√

The ablation experiments were carried out in the same environment as the experimental dataset. The extraction results of the supraspinatus from the images of the test set by each ablation model are shown in Table 3.

**Table 3:** Extraction results of ablation experiments

Model	Pre (%)	IoU (%)	Dice (%)
Baseline	89.03	73.66	84.83
Scheme 1	91.71	75.91	86.3
Scheme 2	89.01	76.85	86.91
Scheme 3	90.24	78.11	87.71
A-DAsppUnet	**92.77**	**83.38**	**90.94**

Bold values indicate the performance of the proposed method over the comparison method

**Fig. 6:**
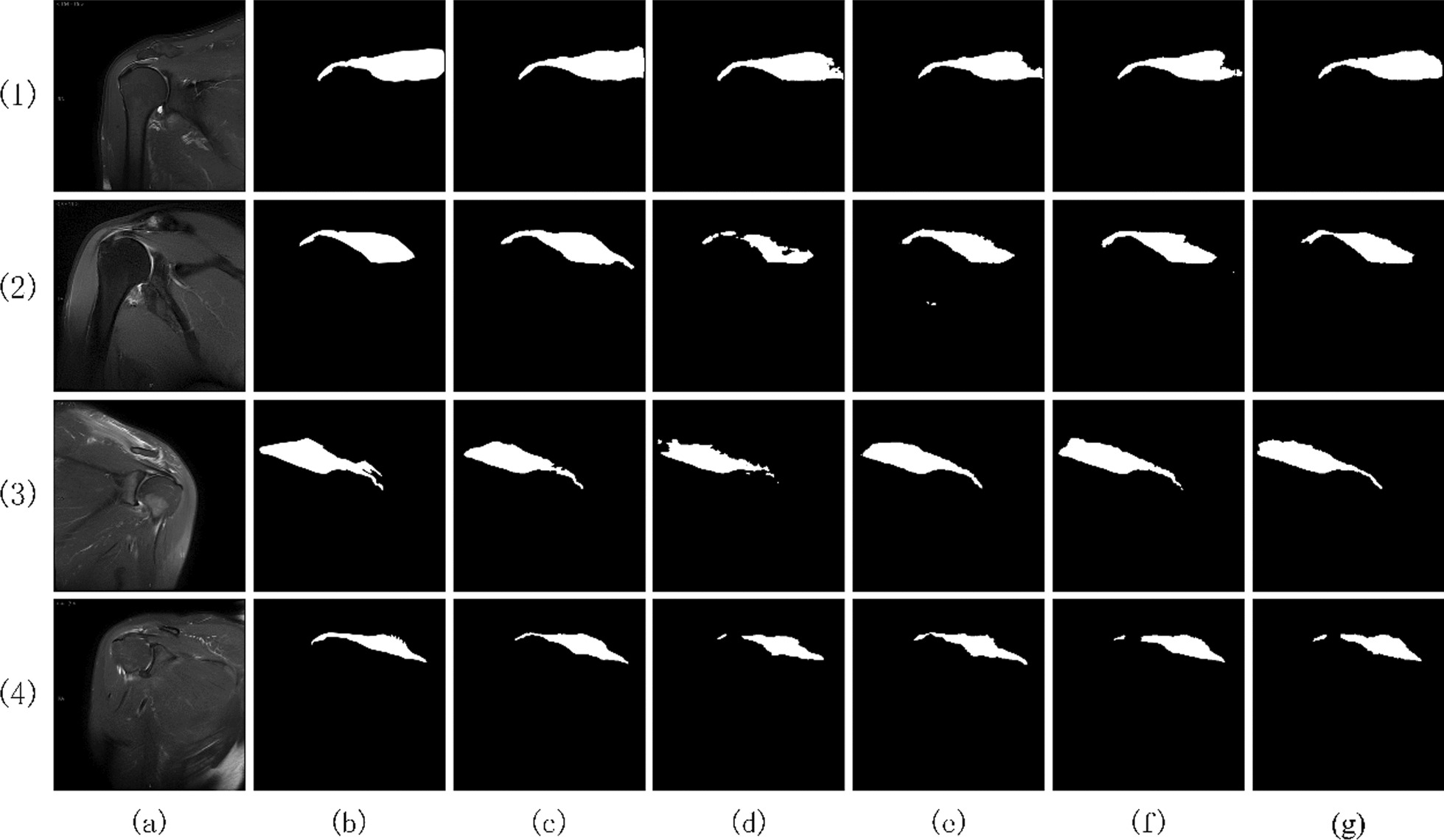
Visualization of ablation model extraction results. **a** Images. **b** Labels. **c** A-DAsppUnet. **d** Baseline. **e** Scheme 1. **f** Scheme 2. **g** Scheme 3

Figure 6 shows the extraction results of the supraspinatus in the test set images of each ablation model. According to the comprehensive table and Fig. 6, the Pre, IoU, and Dice indexes of Scheme 1 increased by 2.68%, 2.25%, and 1.47%, respectively, and the extraction integrity of the supraspinatus was improved by the model. For example, the extraction results of images (2) and (3) are complete, continuous, and clear. In Scheme 2, the channel attention mechanism is added on the basis of Scheme 1, and the IoU and Dice coefficients are increased by 0.94% and 0.61%, respectively. The channel attention mechanism enhances the fusion of important features in the jump connection, thereby improving the accuracy of supraspinatus edge extraction.

In Scheme 3, the DenseASPP module is used as the middle part of connection coding–decoding, and the model performance indexes Pre, IoU, and Dice are increased by 1.21%, 4.45%, and 2.88%, respectively. The DenseASPP module extends the receptive field of the down-sampled feature maps obtained from the encoder without reducing their resolution. It preserves rich feature information and effectively helps the model recognize and extract target regions after up-sampling in the decoder. This module achieved the best results in terms of the integrity, accuracy, and clarity of supraspinatus edge extraction.

## Discussion

With the advancement of medical imaging technology, the quantity and complexity of medical imaging data are continuously increasing. In most cases, even with access to shoulder MRI, nonorthopedic surgeons may find it challenging to identify and diagnose RCTs. In this case, the application of CAD techniques provides support for ensuring high efficiency and accuracy in clinical diagnosis.

Ledley et al. [20] pioneered the field of CAD by building a mathematical model for lung cancer diagnosis. With the emergence of artificial intelligence, CAD has evolved into a DL approach, which has shown great potential and widespread application in image processing and computer vision. DL models have revolutionized the field of medical image analysis by leveraging their ability to extract complex patterns and features from images. Through training on extensive datasets, these models can learn to identify subtle abnormalities, assist in disease diagnosis, and provide valuable insights for clinical work.

DL methods have made significant contributions to medical imaging research, with representative works including brain tumor segmentation [21], lung nodule detection [22], and case image segmentation [23]. In the field of musculoskeletal imaging, accurate imaging diagnosis is crucial, which has spurred the vigorous development of DL techniques. Research in this domain encompasses various areas, such as knee cartilage injury [24], meniscus and ligament tears [25, 26], spinal canal stenosis [27], bone age detection, and osteoporosis diagnosis [28], all of which have achieved fruitful results. In this context, the focus is on shoulder MRI, where the mature technologies primarily concentrate on the segmentation of bony tissues. However, the extraction of imaging features related to musculoskeletal tissue, as well as research on their role in assisting diagnosis, is still under development. Research on robust and accurate algorithms for the segmentation and analysis of these soft tissues in shoulder MRI holds great potential for improving diagnostic accuracy and facilitating treatment planning in orthopedics.

Indeed, DL research based on shoulder MRI has made significant progress. Kim et al. [29] developed a FCN model for the segmentation of the supraspinatus and supraspinatus fossa in the sagittal plane of MRI, which visualizes the degree of supraspinatus atrophy and fatty infiltration. Medina et al. [30] utilized an improved UNet convolutional neural network (CNN) architecture to accurately segment the supraspinatus, infraspinatus, and subscapularis in sagittal plane MRI. Ro et al. [31] employed a CNN-based approach to segment the supraspinatus and supraspinatus fossa. They analyzed the occupation rate of the supraspinatus and utilized an improved Otsu thresholding technique to quantify the extent of fatty infiltration in the supraspinatus. These studies, focusing on sagittal plane of shoulder MRI, enable physicians to accurately assess the degree of supraspinatus atrophy and fatty infiltration and predict the effectiveness of rotator cuff repair surgery.

However, RCTs are primarily categorized as tendinopathy. Only knowing the atrophy and fatty infiltration of the supraspinatus has limited clinical significance. Therefore, the current trend is to study the tendons themselves. Yao et al. [32] employed a three-stage pipeline consisting of ResNet, UNet, and CNN to perform screening, segmentation, and binary classification (tear or no tear) of supraspinatus images. Hess et al. [33] utilized nnUNet to segment both the bony structures (humerus and scapula) and the rotator cuff on a shoulder MR T1-weighted sequence. Lin et al. [34] used four parallel 3D ResNet50 convolutional neural network architectures to detect and classify RCTs based on tear types.

This paper focuses on the supraspinatus and constructs an A-DAsppUnet model, attempting to segment the supraspinatus in the same MRI sequence. Compared with the results of other segmentation models, the proposed model has better segmentation accuracy and performance. It validated the feasibility of using DL methods for segmenting the rotator cuff, and the results provide a reference for clinical treatment and surgical planning in this paper.

However, it is important to acknowledge the limitations of the study. Although the data volume was increased through data augmentation, the experimental data in this study are still not abundant, the prediction results may have minor errors in displaying subtle tears. The training set images only outline the contour of the supraspinatus, so the model prediction results cannot reflect internal injuries and tendon quality. Full-thickness tears mean continuous interruptions of the rotator cuff, leading to significant errors in the segmentation of the tendon stump. Additionally, the boundaries between the supraspinatus and adjacent muscles, such as the trapezius, appear unsatisfactory due to the similarity in pixel grayscale values on MRI. Furthermore, it excluded cases affected by other shoulder diseases, limiting the clinical utility of this model. Addressing the aforementioned issue and expanding the dataset to encompass a broader range of cases would enhance the model's generalization capability.

## Conclusion

In this study, it aimed to investigate the effectiveness of DL models for the extraction of the supraspinatus from shoulder MRI. An improved DL network model was designed, and extensive experiments were carried out on self-constructed supraspinatus dataset.

The experimental results demonstrated that the proposed improved DL model has excellent performance in extracting the supraspinatus the coronal plane of shoulder MRI. The model achieved a high segmentation accuracy with Dice coefficient, precision, and IoU of 0.91, 0.99, and 0.83, respectively. These results indicate that the DL method is capable of accurately segmenting the supraspinatus in shoulder MRI.

Furthermore, the analysis revealed several advantages of the model. The proposed model demonstrates robustness to variations in the position and shape of the supraspinatus. It exhibits resistance to noise interference and achieves high-quality and complete extraction. Compared to traditional image processing techniques, the model outperforms them and shows greater potential in clinical research and applications.

DL-based image segmentation has several advantages compared to the detection and classification of RCTs. Image segmentation offers more detailed information and supports quantitative analysis. It accurately delineates structures or abnormalities at the pixel level, enabling precise localization and providing rich anatomical and pathological details. Therefore, DL-based image segmentation is better suited for handling complex scenarios and personalized medical interventions.

In summary, the research demonstrates the effectiveness of DL models in extracting the supraspinatus from the coronal plane of shoulder MRI. This validates the experimental value and practical significance of DL methods in assisting medical decision-making. Future studies can make breakthroughs by continuously exploring attention mechanisms and multi-scale structures, such as dilated convolutions, and utilizing high-quality data from multiple centers, fully harnessing the potential of DL methods in musculoskeletal imaging.

## Data Availability

The data and code that support the findings of this study are available from the corresponding author upon reasonable request.

## Abbreviations

RCTs: Otator cuff tears
CAD: Computer-aided diagnosis
DL: Deep learning
A-DAsppUnet: Attention-dense atrous spatial pyramid pooling UNet
DASPP: Dense atrous spatial pyramid pooling
DenseNet: Dense convolutional network
PD: Proton density
FCN: Fully convolution network
SegNet: Semantic segmentation network
BCELoss: Binary cross-entropy loss function
Pre: Precision
IoU: Intersection over union
CNN: Convolutional neural network
